# Bone structural changes after gastric bypass surgery evaluated by HR-pQCT: a two-year longitudinal study

**DOI:** 10.1530/EJE-17-0014

**Published:** 2017-03-13

**Authors:** Vikram V Shanbhogue, René Klinkby Støving, Katrine Hartmund Frederiksen, Stine Hanson, Kim Brixen, Jeppe Gram, Niklas Rye Jørgensen, Stinus Hansen

**Affiliations:** 1 Department of EndocrinologyOdense University Hospital, Odense C, Denmark; 2 Institute of Clinical ResearchUniversity of Southern Denmark, Odense C, Denmark; 3 Center for Eating DisordersOdense University Hospital & Psychiatry of Region Southern Denmark, Odense C, Denmark; 4 Department of EndocrinologyHospital of Southwest Denmark, Esbjerg, Denmark; 5 Department of Clinical BiochemistryResearch Center for Ageing and Osteoporosis, Glostrup, Denmark; 6 OPENOdense Patient data Explorative Network, Odense University Hospital/Institute of Clinical Research, University of Southern Denmark, Odense, Denmark

## Abstract

**Objective, design and methods:**

Roux-en-Y gastric bypass (RYGB) has proved successful in attaining sustained weight loss but may lead to metabolic bone disease. To assess impact on bone mass and structure, we measured a real bone mineral density at the hip and spine by dual-energy X-ray absorptiometry, and volumetric BMD (vBMD) and bone microarchitecture at the distal radius and tibia by high-resolution peripheral quantitative CT in 25 morbidly obese subjects (15 females, 10 males) at 0, 12 and 24 months after RYGB. Bone turnover markers (BTMs), calciotropic and gut hormones and adipokines were measured at the same time points.

**Results:**

After a 24.1% mean weight loss from baseline to month 12 (*P* < 0.001), body weight plateaued from month 12 to 24 (−0.9%, *P* = 0.50). However, cortical and trabecular vBMD and microarchitecture deteriorated through the 24 months, such that there was a 5 and 7% reduction in estimated bone strength at the radius and tibia respectively (both *P* < 0.001). The declines observed in the first 12 months were matched or exceeded by declines in the 12- to 24-month period. While a significant increase in BTMs and decrease in leptin and insulin were seen at 24 months, these changes were maximal at month 12 and stabilized from month 12 to 24.

**Conclusions:**

Despite weight stabilization and maintenance of metabolic parameters, bone loss and deterioration in bone strength continued and were substantial in the second year. The clinical importance of these changes in terms of increased risk of developing osteoporosis and fragility fractures remain an important concern.

## Introduction

Morbid obesity is an increasing health problem and is associated with a substantial increase in mortality rate attributed to a number of co-morbid conditions (1). Conservative treatment with dietary and other lifestyle interventions often prove unsuccessful since an initial weight loss is followed by a renewed, often surplus weight gain (2). A number of surgical procedures and Roux-en-Y gastric bypass (RYGB) in particular, have proved successful in attaining sustained weight loss, lower mortality rates and improve or even resolve some co-morbid conditions such as type 2 diabetes mellitus (3, 4).

The main weight loss after RYGB occurs within the first six to twelve months and body weight then plateaus or slightly increases in the following years (4). Bone mineral density (BMD) in the spine and hip rapidly declines in the first year after surgery which likely represents a skeletal adaptation to the lower body weight (5, 6, 7, 8). The few studies that have assessed long-term bone effects after RYGB indicate that bone loss continues even after weight loss has ceased (9, 10) suggesting that post-RYGB induced metabolic changes may have effects beyond those outplayed in the acute weight loss phase and may thus represent an unintended effect of the procedure. The potential mechanisms include a diminished gastric and short bowel area available for vitamin and nutritional uptake causing lower calcium absorption and vitamin D insufficiency leading to secondary hyperparathyroidism. Also, the levels of a number of intestinal hormones and cytokines are altered. The extent to which such changes have long-term deleterious effects on bone health are unknown (11).

The valid assessment of bone status in obese subjects using dual-energy X-ray absorptiometry (DXA) are diminished by technical constraints where soft-tissue artefacts may cause unpredictable impacts on BMD (11). Also DXA has lower precision with increasing body mass index (12). In obese subjects, these constraints appear to be less pronounced when BMD is measured based on three-dimensional imaging using quantitative computed tomography (QCT) in comparison to DXA (13). Further, with the use of newer 3D imaging systems with improved resolution, such as high-resolution peripheral QCT (HR-pQCT), the differential effects in various bone compartments may be disentangled with the assessment of geometry, volumetric BMD (vBMD) and microarchitecture in both the cortical and trabecular compartments in radius and tibia (14). A few studies have evaluated bone changes after bariatric surgery using this technique. In a cohort of patients treated with RYGB, gastric banding or sleeve gastrectomy, Stein *et al.* (6) found declines in cortical thickness and vBMD at the tibia 12 months post-surgery. Previously, we have shown substantial declines in indices of both cortical and trabecular BMD and microarchitecture as well as estimated strength at the tibia while only few changes at the radius 12 months after RYGB (15). Similar results were reported in a study by Yu *et al*. (10) that found similar changes in tibia after 12 months and reported continued bone loss in both radius and tibia assessed 24 months after surgery.

In this two-year extension study, we report changes in HR-pQCT-based compartmental geometry, vBMD, microarchitecture and estimated strength, as well as biochemical markers of bone turnover, calciotropic hormones and adipokines after RYGB. We hypothesized that bone loss would be more pronounced at the tibia compared to the radius and that the changes from baseline to 24 months compared to 12 months would generally be mitigated as body weight plateaued.

## Subjects and methods

### Participants

**Figure 1:**
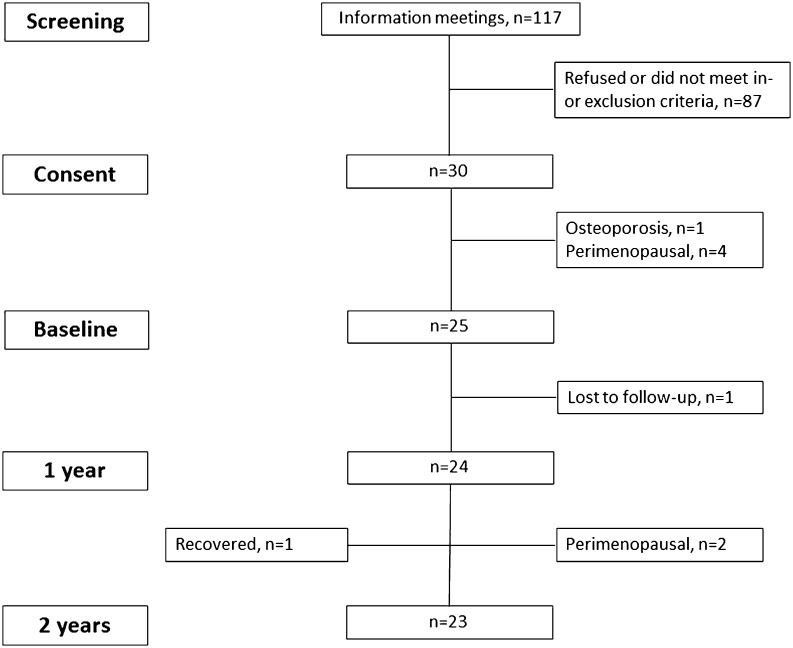
Flowchart of inclusion.

We recruited 25 obese subjects eligible for RYGB (Fig. 1) using Danish guideline criteria (age >25 years and body mass index (BMI) >50 kg/m^2^ or BMI >35 kg/m^2^ along with at least one obesity-related complication) and planned for surgery, from the Department of Endocrinology, Odense University Hospital and the Department of Endocrinology, Hospital of Southwest Denmark between October 2011 and August 2012 as previously described (16). In brief, subjects were excluded if they were non-ambulatory, pregnant, perimenopausal or had metabolic bone disease including osteoporosis or used medications with known effects on bone metabolism at baseline. Subjects had study visits at 12 and 24 months of follow-up and participants were advised dietary recommendations as per local hospital guidelines. The study was approved by the Regional Scientific Ethical Committee for Southern Denmark. Informed consent was obtained from each subject after full explanation of the purpose and nature of all procedures used and the study was performed according to the guidelines of the Declaration of Helsinki.

### DXA

Areal BMD was measured at the total hip and lumbar spine (L1–L4) using DXA (Hologic, Waltham, MA, USA) at baseline, one and two years after surgery. The coefficient of variation (CV) is 1.5% at both the spine and hip in our unit. Also, a whole body DXA scan was performed to measure total fat and lean body mass. Quality control was maintained with daily measurement of a Hologic DXA spine phantom.

### HR-pQCT

Geometry, volumetric BMD (vBMD) and microarchitecture of the distal radius and tibia were assessed using HR-pQCT (XtremeCT, Scanco Medical, Switzerland) at months 0, 12 and 24. The standard protocol for image acquisition and analyses was applied as described previously (14), and a detailed description of the measurement protocol at our centre is described in (15). Quality control was performed daily and up to three scans at each site were obtained to get optimum image quality. Post-acquisition image grading was performed by one author (STH). An automatic common region matching procedure based on variation in bone cross-sectional area was applied to ensure that only volumes common to scans obtained at all visits were used for the extraction of parameters. An additional image evaluation was performed to further assess details of the cortical bone compartment as described previously (17). Also, radius and tibia bone failure load was estimated using a finite element analyses solver (Finite Element Analysis Software version 1.15, Scanco Medical) (18). In our unit, the CV for geometry, vBMD and microarchitecture measures at the radius ranges from 0.3 to 1.7%, 0.6 to 0.9% and 3.9 to 7.2% respectively, and the CV for geometry, vBMD and microarchitecture measures at the tibia ranges from for 0.2 to 1.2%, 0.4 to 0.6% and 3.7 to 7.0% respectively, as detailed elsewhere (19). The CVs for estimated failure load were 1.7 and 1.2% at the radius and tibia respectively.

### Biochemical analyses

A fasting blood sample was obtained at each visit. We measured parathyroid hormone (PTH) (Immunolite 2000, Siemens), 25-OH-vitamin D (Cobas e411, Roche Diagnostics), follicle stimulating hormone (FSH) and luteinizing hormone (LH) (both AutoDelfia, Perkin Elmer). For these analyses the coefficient of variation (CV) ranged from 4 to 6%. Adiponectin and leptin were measured (Bio-Plex Pro assay, Bio-Rad Laboratories) as were procollagen type I amino-terminal propeptide (P1NP) and C-terminal telopeptide of type 1 collagen (CTX-I) ((iSYS, Immunodiagnostic Systems) with CV for these analyses ranging from 8 to 10%. Last, insulin (Elecsys 2010, Roche Diagnostics) was measured with a CV of 3.5%.

### Statistical analyses

We report data as mean ± s.d. or median (interquartile range) as appropriate. Repeated measures ANOVA with time-wise comparisons were performed to compare values between the baseline, one and two year’s visits. Spearman’s correlation analysis was performed to assess predictors of bone loss (baseline age and weight as well as changes in weight, lean mass, ratio of lean mass to fat mass and biochemical indices from baseline to two years). As this is the first study exploring the predictors of changes in bone microarchitecture post-RYGB, our intention was to report our findings in a broader context and generate hypotheses. Hence, we did not formally adjust for multiple comparisons, but have interpreted our findings cautiously. A *P*-value below 0.05 was considered significant. All statistical analyses were performed using Stata Statistical Software release 11.0 (StataCorp LP).

## Results

### Study population characteristics

Of the 25 subjects at baseline, one subject missed the one year visit but was examined at the remainder time points and thus all 25 subjects were available for the two-year study. Two female participants were excluded since they entered menopause during the study period as judged from debut of irregular periods and a rapid rise in gonadotrophins. Therefore, a total of 23 subjects (one post- and 13 pre-menopausal women, 9 men) were included in the two-year analyses (Table 1). Six participants had type 2 diabetes and all received anti-diabetic treatment at baseline whereas this was required in only one patient at the one- and two-year visits. One participant had type 1 diabetes.

**Table 1:** General characteristics, biochemistry and DXA results in patients at baseline, one and two years after gastric bypass surgery. Data are means ± s.d. or median (range) as appropriate.

	**Baseline** (*n* = 23)	**One year** (*n* = 22)	**% Change vs baseline**	**Two years** (*n* = 23)	**% Change vs baseline**
Age (years)	42.6 ± 7.8	43.8 ± 7.8	–	44.7 ± 7.8	–
Sex (female/male)	14/9	14/9	–	14/9	–
Height (cm)	172 ± 9	–	–	–	–
Weight (kg)	124 (108, 144)	95 (81, 112)^b^	−24.1	94 (79, 110)^b^	−25.2
Body mass index (kg/m^2^)	42 (38, 47)	31 (28, 37)^b^	−24.1	31 (27, 36)^b^	−25.2
Biochemistry					
PTH (pmol/L)	5.1 (3.1, 7.3)	4.2 (2.7, 6.5)	−9.7	4.8 (3.5, 6.4)	−6.5
25-OH-vitamin D2 + D3 (nmol/L)	34 ± 16	59 ± 15^b^	109.2	93 ± 32^b,d^	240.4
P1NP (µg/L)	36 (29, 45)	80 (62, 93)^b^	113.6	78 (63, 103)^b^	112.9
CTX-1 (µg/L)	0.42 ± 0.22	1.12 ± 0.48^b^	166.7	0.63 ± 0.35^a,d^	40.0
Adiponectin (mg/L)	3.6 (3.0, 6.2)	7.4 (6.1, 11.5)^b^	109.6	5.9 (4.3, 8.4)^b,d^	62.0
Leptin (ng/mL)	42 (27, 64)	10 (5, 29)^b^	−70.0	9 (5, 11)^b^	−77.2
Insulin (pmol/L)	151 (117, 212)	48 (35, 56)^b^	−68.0	40 (30, 51)^b^	−73.5
DXA					
Total hip aBMD (g/cm^2^)	1.11 ± 0.12	1.02 ± 0.14^b^	−8.2	0.99 ± 0.15^b,c^	−10.5
Lumbar spine aBMD (g/cm^2^)	1.08 ± 0.14	1.04 ± 0.15^b^	−3.5	1.03 ± 0.15^b^	−5.3
Total fat mass (kg)	56 ± 12	34 ± 11^b^	−40.1	35 ± 10^b^	−38.0
Total lean mass (kg)	70 ± 14	61 ± 14^b^	−13.1	61 ± 14^b^	−13.0
Total fat percent (%)	46 (38, 50)	37 (30, 42)^b^	−21.0	38 (29, 43)^b^	−19.1

a and b indicate significance of differences between the one year or two year values compared to baseline values and c and d indicate significance of differences between the two-year values compared to one-year values.

a *P* < 0.05, ^b^*P* < 0.001, ^c^*P* < 0.05, ^d^*P* < 0.001.

aBMD, areal bone mineral density; CTX-1, C-terminal telopeptide of type 1 collagen; DXA, dual energy X-ray absorptiometry; HRT, hormone replacement therapy; PTH, parathyroid hormone; P1NP, procollagen type 1 amino-terminal propeptide.

### Body weight

A median weight loss of 32 kg (range 3–59) was obtained from baseline to month 12 (−24.1%, *P* < 0.001) whereas body weight then levelled out and did not change from month 12 to 24 (−0.9%, *P* = 0.50).

### Biochemistry

Serum PTH remained stable throughout the 24-month study (Table 1). However, there was a persistent and significant increase in 25-OH-vitamin D at follow-up month 12 and month 24 (109.2 and 240.4% respectively, both *P* < 0.001). In comparison to baseline, there was a marked increase in P1NP and CTX1 after 12 months (113.6 and 166.7% respectively, both *P* < 0.001), and while P1NP did not change and CTX1 decreased from month 12 to 24 (−43.8%, *P* < 0.001), the median P1NP and CTX1 remained 112.9% (*P* < 0.001) and 40.0% (*P* < 0.05) above baseline at 24 months respectively. Similarly, we found an initial 109.6% increase in median adiponectin levels after 12 months (*P* < 0.001), that although decreased by 25.3% during the 24-month follow-up in comparison to the 12-month values (*P* < 0.001), remained 62.0% above the baseline value (*P* < 0.001). On the other hand, serum leptin and insulin levels displayed a marked decrease after 12 months (−70.0 and −68.0% respectively, both *P* < 0.001), that stabilized from month 12 to 24 but persisted to be significantly lower than baseline levels (−77.2 and −73.5% respectively, both *P* < 0.001).

### DXA

There was a significant decrease in mean total hip aBMD and spine aBMD after 12 (−8.2 and −3.5% respectively, both *P* < 0.001) and 24 months (−10.5 and −5.3% respectively, both *P* < 0.001) in comparison to baseline. However, while the mean total hip aBMD displayed persistent decrease, spine aBMD did not change from 12 to 24 month (−2.1%, *P* < 0.05 and −1.5%, *P* = 0.06 respectively). Further, we found a significant fall in total fat mass, fat percent and lean mass from baseline to month 12 (all *P* < 0.001), which stabilized and did not change from month 12 to 24 (Table 1).

### HR-pQCT

**Figure 2:**
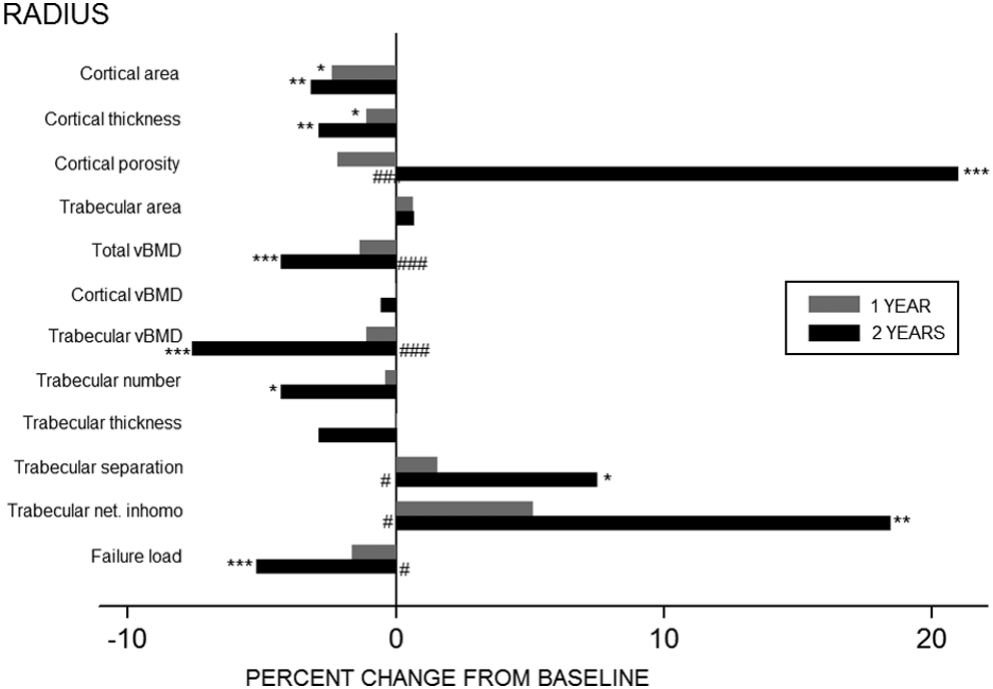
Percent change in HR-pQCT parameters in radius one (grey bars) and two years (black bars) after gastric bypass surgery. *P < 0.05, **P < 0.01, ***P < 0.001 vs baseline ^#^*P* < 0.05, ^##^*P* < 0.01, ^###^*P* < 0.001 vs one-year value. Trabecular net. inhomo, trabecular network inhomogeneity.

Bone geometry, vBMD, indices of bone microarchitecture and estimated bone strength deteriorated throughout the study period (Table 2). At the radius, in comparison to baseline measurements, cortical area declined significantly (−3.2%, *P* < 0.01) whereas trabecular area did not change at month 24 (0.7%, *P* = 0.07). Total vBMD declined by 4.3% from baseline to month 24 (*P* < 0.001) and was predominantly contributed by a 7.6% fall in trabecular vBMD (*P* < 0.001). Alongside the decline in trabecular vBMD there was a loss of entire trabeculae leading to a reduction in trabecular number (−4.3%, *P* < 0.05), increase in trabecular separation (7.5%, *P* < 0.05) and a greater trabecular network inhomogeneity (18.5%, *P* < 0.01). Although there was no significant change in cortical vBMD 24 months post-RYGB, there was a 2.9% decrease in cortical thickness (*P* < 0.01) and a 21.0% increase in cortical porosity (*P* < 0.05). Estimated failure load declined by 5.2% throughout the study period (*P* < 0.01). Of note, except for cortical area and cortical thickness where the rate of decline was most prominent at month 12, with stabilization from month 12 to 24, the rate of loss of vBMD and deterioration of microarchitectural parameters were most pronounced in the second year post-RYGP (Fig. 2).

**Table 2:** Bone geometry, vBMD, microarchitecture and estimated strength using HR-pQCT in patients at baseline and one and two years after gastric bypass surgery. Data are means ± s.d. or median (range) as appropriate.

	**Baseline** (*n* = 23)	**One year** (*n* = 22)	**Two years** (*n* = 23)
Radius			
Cortical area (mm^2^)	69 ± 14	68 ± 15^a^	67 ± 15^b^
Cortical thickness (mm)	0.94 ± 0.15	0.92 ± 0.16^a^	0.91 ± 0.15^b^
Cortical porosity (%)	1.38 (0.79, 2.44)	1.37 (0.91, 1.83)	1.78 (0.94, 2.64)^d^
Trabecular area (mm^2^)	244 ± 89	247 ± 91	245 ± 89
Total vBMD (mg/cm^3^)	354 ± 47	351 ± 45	339 ± 46^c,f^
Cortical vBMD (mg/cm^3^)	906 ± 51	904 ± 52	900 ± 50
Trabecular vBMD (mg/cm^3^)	175 ± 43	176 ± 40	162 ± 44^c,f^
Trabecular number (1/mm)	2.20 (1.82, 2.41)	2.19 (2.00, 2.26)	2.09 (1.72, 2.34)^a^
Trabecular thickness (mm)	0.066 (0.059, 0.080)	0.070 (0.064, 0.076)	0.063 (0.060, 0.075)
Trabecular separation (mm)	0.383 (0.345, 0.488)	0.390 (0.373, 0.424)	0.398 (0.365, 0.538)^a,d^
Trabecular network inhomogeneity (mm)	0.154 (0.131, 0.203)	0.161 (0.144, 0.170)	0.163 (0.140, 0.239)^b,d^
Estimated failure load (N)	5000 ± 1450	4993 ± 1424	4757 ± 1461^c,d^
Tibia			
Cortical area (mm^2^)	161 ± 34	157 ± 32^c^	149 ± 34^c,f^
Cortical thickness (mm)	1.46 ± 0.22	1.41 ± 0.20^c^	1.34 ± 0.22^c,f^
Cortical porosity (%)	5.45 ± 2.4	5.79 ± 2.8	6.56 ± 3.4^c,e^
Trabecular area (mm^2^)	622 ± 152	628 ± 157^a^	630 ± 155^c^
Total vBMD (mg/cm^3^)	341 ± 43	334 ± 47^a^	318 ± 53^c,f^
Cortical vBMD (mg/cm^3^)	893 ± 42	881 ± 52^a^	871 ± 57^c,e^
Trabecular vBMD (mg/cm^3^)	193 ± 38	189 ± 38	179 ± 40^c,f^
Trabecular number (1/mm)	2.32 (2.09, 2.56)	2.22 (1.88, 2.50)^c^	2.18 (1.89, 2.42)^c^
Trabecular thickness (mm)	0.071 (0.062, 0.075)	0.074 (0.064, 0.082)	0.073 (0.063, 0.080)
Trabecular separation (mm)	0.354 (0.323, 0.419)	0.369 (0.332, 0.473)^b^	0.386 (0.340, 0.448)^c^
Trabecular network inhomogeneity (mm)	0.142 (0.120, 0.182)	0.152 (0.131, 0.211)^a^	0.159 (0.130, 0.200)^c^
Estimated failure load (N)	13 108 ± 2588	12 933 ± 2722	12 255 ± 2814^c,f^

a, b and c indicate significance of differences between the one-year or two-year values compared to baseline values and d, e and f indicate significance of differences between the two-year values compared to one-year values.

a *P* < 0.05, ^b^*P* < 0.01, ^c^*P* < 0.001, ^d^*P* < 0.05, ^e^*P* < 0.01, ^f^*P* < 0.001.

vBMD, volumetric bone mineral density.

**Figure 3:**
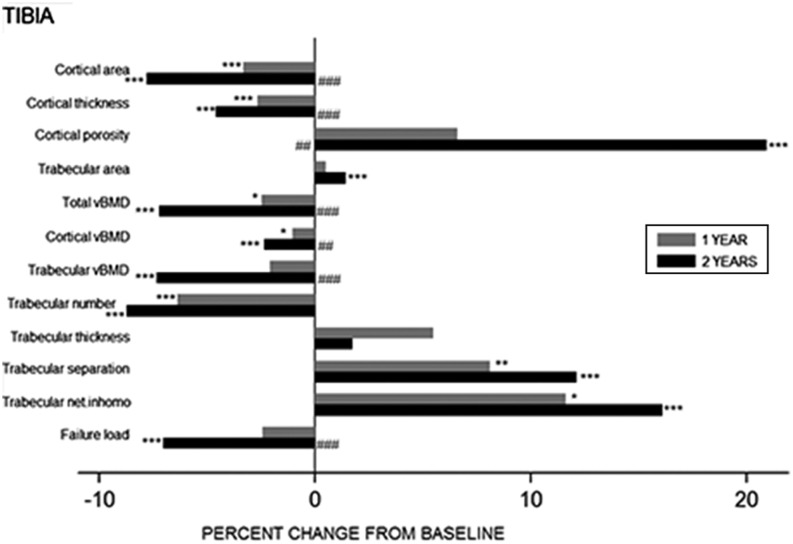
Percent change in HR-pQCT parameters in tibia one (grey bars) and two years (black bars) after gastric bypass surgery. *P < 0.05, **P < 0.01, ***P < 0.001 vs baseline ^#^*P* < 0.05, ^##^*P* < 0.01, ^###^*P* < 0.001 vs one-year value. Trabecular net. inhomo, trabecular network inhomogeneity.

At the tibia, there was a persistent decrease in cortical area (−7.8%, *P* < 0.001) and a reciprocal increase in trabecular area (1.4%, *P* < 0.05) at month 24 from baseline. Total vBMD progressively declined throughout the study period (−7.2%, *P* < 0.001). This loss in total vBMD was due to both, a decline in trabecular vBMD (−7.4%, *P* < 0.001) with reduced trabecular number (−8.7%, *P* < 0.001) , increased trabecular separation (8.0%, *P* < 0.001) and greater trabecular network inhomogeneity (16.1%, *P* < 0.001); and a reduction in cortical vBMD (−2.4%, *P* < 0.01) due to thinning of the cortex (−4.6%, *P* < 0.001) and an increase in cortical porosity (20.9%, *P* < 0.01). These changes lead to a compromise in the estimated failure load 2-year post-RYGB (−7.0%, *P* < 0.001). Overall, the changes observed in trabecular geometrical and microarchitectural parameters were maximal in the first 12 months and then plateauing from month 12 to 24, whereas declines in cortical area, total and cortical vBMD generally progressed throughout the 2-year study period, and deteriorations in trabecular vBMD and estimated failure load were most conspicuous in the 12- to 24-month period (Fig. 3).

### Predictors of bone loss

While baseline characteristics such as age and weight, change in weight from baseline to month 12 and change in the ratio of lean mass to fat mass were not the predictors of 24-month changes in DXA- or HR-pQCT-based bone parameters, changes in lean mass correlated with changes in total hip aBMD (*rho* 0.45, *P* = 0.03) and tibial cortical thickness (*rho* 0.47, *P* = 0.02), trabecular area (*rho* −0.44, *P* = 0.04) and trabecular network inhomogeneity (*rho* −0.47, *P* = 0.02). None of the changes in biochemical parameters from baseline to 24 months correlated with changes in DXA or HR-pQCT measures except a negative correlation between changes in adiponectin, CTX1, P1NP and changes in total hip aBMD (*rho* −0.44, *P* = 0.045; *rho* −0.59, *P* = 0.004 and *rho* −0.45, *P* = 0.038 respectively), changes in insulin and changes in estimated failure load (*rho* −0.48, *P* = 0.02) and positive correlation between changes in P1NP and cortical porosity (*rho* −0.62, *P* = 0.002) at the radius, and negative correlation between changes in P1NP and total and cortical vBMD and estimated failure load (*rho* −0.44, *P* = 0.039; *rho* −0.44, *P* = 0.039 and *rho* −0.52, *P* = 0.013 respectively) at the tibia.

## Discussion

In contrast to our hypothesis, the findings from this study indicate a persistent decline in BMD and a progressive deterioration in bone microarchitecture and estimated strength at both weight bearing and non-weight bearing skeletal sites in the second year after Roux-en-Y gastric bypass. This continued and excessive bone loss persisted despite the attainment of steady-state in weight, fat and lean body mass, adipokines such as leptin and gut hormones such as insulin one year after surgery. There was no change in calcium or parathyroid hormone during the course of the study probably because of abundant vitamin D due to high daily supplementation doses. Overall, these results lend credence to the notion that adaptation of bone is a slow, ongoing process that continues after the stabilization of metabolic parameters and body weight such that attainment of skeletal equilibrium lags metabolic equilibrium.

Unlike the earlier findings of significant compromises in trabecular and cortical compartments at the tibia with only minimal changes at the radius 12 months post-RYGB (15), extension of the current study into the second year demonstrated significant changes at both the radius and tibia. There were significant declines in total vBMD at both peripheral sites. While at the radius, this was manifest predominantly in the trabecular compartment with the loss of trabecular vBMD and microarchitectural deterioration, bone loss at the tibia was due to declines in both trabecular and cortical vBMD due to the loss of entire trabecular struts and a thinner, more porous cortex respectively. The magnitude of these changes resulted in significantly reduced estimated bone strength at both the skeletal sites examined. These findings are consistent with the previous two-year follow-up study by Yu *et al*. (10), who demonstrated an identical pattern of findings with predominantly trabecular bone loss at the radius and both, cortical and trabecular bone loss at the tibia, causing a reduced estimated bone strength at both sites. Similar to our study, Yu *et al*. (10) observed that the declines observed in BMD, microarchitecture and estimated bone strength in the first 12 months were matched or exceeded by declines in the 12- to 24-month period after gastric bypass.

The exact mechanisms underlying bone loss after RYGB are still unclear and probably multifactorial. While adaptive remodelling in response to mechanical unloading (20), failure to maintain lean muscle mass (21) and mal-absorption leading to changes in calciotropic hormones (22) are frequently cited, postoperative neuro-hormonal and gut hormone alterations (23) also offer potential preliminary hypotheses. Adipose tissue has been increasingly recognized to harbour hormones (leptin, adiponectin and oestrogens, among others) with a substantial impact on bone metabolism (24). While there is some evidence that leptin is positively associated and adiponectin is negatively associated with aBMD (25, 26, 27), and the decrease in leptin and increase in adiponectin as seen after RYGB (28) would be anticipated to result in a decrease in bone mass, the ultimate effect of these adipokines on bone post-RYGB remains to be elucidated. Hyperinsulinaemia has been associated with a superior vBMD and microarchitecture (29). Thus a fall in insulin levels post-RYGB would be expected to negatively impact bone homeostasis. Although we did find significant decreases in leptin and insulin and increase in adiponectin in our study, we were unable to demonstrate consistent correlations between the changes in these hormones and the change in various bone parameters probably because of the limited sample size as well as the fact that deterioration in bone parameters was persistent throughout the 24 months study period while peak changes in adipokines and insulin was at month 12 followed by plateauing or no change from month 12 to 24.

Consistent with previous studies (6, 10), we found that changes in the cortical compartment were most conspicuous at the tibia whereas cortical vBMD was preserved at the radius. This disparate involvement of weight bearing and non-weight bearing skeletal sites in post gastric bypass patients may be caused by a complex interaction between biomechanical strains that are modulated by ambient oestrogen concentration in the bone microenvironment. As emphasized by Frost *et al*. (30) , chronically lowered strain levels, as seen in weight bearing bones post-RYGB, will induce a disuse mode of bone remodelling with an increased in bone turnover, predominantly at the endocortical surface. Reductions in serum oestrogen levels have been demonstrated in premenopausal women (31) and men (32) after bariatric surgery and there is accumulating evidence indicating that oestrogen has a more dominant role on the mature male skeleton (33). According to the threshold effect of bioavailable estradiol on cortical bone, as described by Khosla *et al*. (33), non-weight bearing bones such as radius may have a lower sensitivity to oestrogen and thus a high threshold to withstand low oestrogen levels. Thus, the lesser impact of changes in mechanical loads on the radius coupled with the possibility that circulating levels of oestrogen in post-RYGB patients may be sufficiently high to maintain cortical integrity at the radius but not at the tibia is a potential explanation for the more marked loss of cortical bone at tibia compared to radius.

In our study, we found marked increases in bone turnover markers at 12 months that remained elevated, although less so, up to 24 months postoperatively despite the absence of secondary hyperparathyroidism. While this was in accordance with previous findings (10, 34), the increase in bone resorption markers exceeded levels typically observed during menopause transition (35). This, coupled with the incessant deterioration in vBMD and architecture after weight has stabilized, has led to the concern that patients are at an increased risk for osteoporosis and related future fracture post-bariatric surgery. The prevalence of osteopenia/osteoporosis after bariatric surgery is controversial with some studies suggesting lower BMD than expected (36) and others finding no difference compared with age- and post-bariatric body mass index-matched controls (9, 37). Similarly, the risk of fractures in a bariatric surgery population is as yet unclear. While Lalmohamed *et al.* (38) reported no significant increase in fracture risk after three to five years following any bariatric surgery procedure, Lu *et al*. (39) showed increased fracture risks over a 12-year follow-up period restricted to mal-absorptive procedures such as gastric bypass but not restrictive procedures. On the other hand, Rousseau *et al*. (40) reported a higher likelihood of fractures only with biliopancreatic diversion but not RYGB or other procedures. Whether the increased fracture risk is a direct consequence of the type of bariatric surgery or an altered risk of falls due to changes in body composition or is reflective of an increased fracture incidence related to the underlying obesity and other comorbidities remains to be determined. Indeed, the paucity of fracture and osteoporosis incidence data raises questions about whether the marked bone loss after bariatric surgery is a physiological adaptation to skeletal unloading or represents a pathophysiological process that may continue after weight stabilization inducing ongoing bone loss and increased bone fragility.

This study has some limitations including the lack of a nonsurgical control group to account for the age-related changes in bone loss, absence of data on physical activity, which may have played a role in the observed changes, as well as absence of biochemical measurements of other gut hormones that potentially affect bone metabolism. The lack of prospective studies examining changes in bone microarchitecture after gastric bypass surgery, at the time that this study was initiated, limited an adequate power calculation. Thus, although the relatively small number of premenopausal women and men in the study limited statistical power and precluded the generalizability of our results to postmenopausal women, the concordance of our results with previous HR-pQCT studies suggest otherwise (6, 10).

In summary, we found persistent declines in volumetric BMD and compromised microarchitectural integrity and estimated bone strength at the appendicular skeleton in the second year after RYGB surgery. This skeletal deterioration was progressive despite weight stabilization and maintenance of fat and lean mass and various other metabolic parameters. Future long-term studies are required to verify continued bone loss after bariatric surgery and evaluate the long-term effects on the skeleton in terms of increased risks of developing osteoporosis and fragility fractures.

## Declaration of interest

The authors declare that there is no conflict of interest that could be perceived as prejudicing the impartiality of the research reported.

## Funding

The study was supported by a grant from the Municipality Region of Southern Denmark. The study was supported by a grant from the Municipality Region of Southern Denmark and acknowledge the University Library of Southern Denmark for their support in publishing the article.

## Authors contribution statement

Study design was done by K B, R S, S H, K H F and S H O. Study conduct was done by K H F, S H O and S H. Data collection was done by S H, K H F, S H O, N R J and J G. Data analysis was done by V S and S H. Data interpretation was done by S H and V S. Drafting manuscript was done by S H and V S. Revising the manuscript content was done by V S, R S, K H F, S H O, K B, J G, N R J and S H. Approving the final version of the manuscript was done by V S, R S, K H F, S H O, K B, J G, N R J and S H.
